# Puerperal Insanity

**Published:** 1874-12-01

**Authors:** W. W. Godding


### PUERPERAL INSANITY.

By W. W. Godding, M.D.

THE vagaries of lunacy, the varying phases of mental delusion, are interesting enough as a diversion, but are of very little concern to the practitioner. That "It pleased God to form poor Ned a thing of idiot mind" hardly arrests our attention beyond the passing wonder why He made him at all; but the question, What can I do with puerperal insanity? may any hour in the day become a practical one to be decided by any one of us. Given a case: what will you do with it? Cases of puerperal mania in the great majority of instances either die within the first two or three weeks from exhaustion with typhoid symptoms, or recover somewhat rapidly, the excitement subsiding at the end of a few days or weeks, rarely continuing months. The exceptional cases neither die nor recover, but pass into chronic dementia. In the onset of the disease there are usually some slight premonitions of a wandering mind, but the nurse does not always remark them, and the explosion follows so soon that an outbreak of violence or destructiveness or obscenity may be the first intimation of what has happened. You find your patient up, walking about the room, or held in bed by two or three strong women, or, it may be, she is lying still, tearing her clothes, swearing, or pouring out a stream of obscenity so foul that you wonder how in her heart of hearts such phrases ever found lodgement. Now what will you do with her? Many answer by promptly sending the case to a hospital, which I have no doubt is very often the best and only thing that can be done, though I have wondered if my considerable experience with typhoid exhaustion had any connection with this promptness. It is certainly to be gravely considered, whether in the first few days after confinement the risk of removal to a hospital at any considerable distance does not more than counterbalance any possible greater benefit to be derived from hospital treatment. Consider, too, before you send to the hospital, that that is a step which once taken can never be recalled out of her life. Treat the case at home, and should it terminate fortunately, the excitement subsiding in a short time, the memory of it in the minds of friends will be of a sickness with some delirium, a little queer as women often are after confinement. Send to the hospital, and, though recovery is rapid and satisfactory, and the woman herself has rather a pleasant recollection of her convalescence, as is generally the case when the recovery is complete, still, she has been insane, and this is never forgotten by her friends or her children; henceforward there is a certain dread of what may be in the future, a skeleton in the closet, not mentioned but always there.

So, the circumstances and condition of the patient justifying, you decide to attempt the treatment at home. You want good nurses who will not gossip, and who are not afraid. The points to be gained are rest in bed, sleep, nutrition. But your patient will not stay in bed; then make her do so. Do not wear out her strength and the patience of your nurses; provide a strong brown linen waist, made full in the bosom, fastening behind, with the sleeves closed at the end and prolonged a yard beyond the hands, then you have something soft and strong that can be tied together behind the back; sometimes you will not need to keep it tied; sometimes, even when tied, she will keep struggling out of bed, and it is a constant exertion for the nurse to keep her in; then pass sheets under the arms and lash her to the bed. Have no nonsense about the looks of the thing; here is one of the cases where "the life is more than raiment." Remember, it is a woman's existence you are trying to save, that typhoid exhaustion is waiting for you if you let her wear out her strength. Perhaps when fairly secured in bed she will go to sleep; that is the best possible result if it comes without hypnotics; if not, perhaps food will bring it. There is an imperative demand for a good supply of easily assimilated nourishment. The patient usually takes it irregularly, but tact on the part of the nurse will generally insure its being taken without forced feeding. Milk I have found about as well taken as anything, which reminds me to say here, entirely out of connection, that you need have little concern about the milk in the breasts; a broken breast is the rarest event in puerperal mania; conservative nature closes this drain on the system at once without your interference.

But you do not get sleep with the administration of food, or from the horizontal position in bed; what then? You have bromide of potassium, chloral hydrate, morphia, including subcutaneous injection of the latter. I would try them in full doses in the order I have named. If they succeed, and they often will partially at least, well; if not, do not overpower the strength by cumulative doses of narcotics, they will not generally give what you are seeking; but darken your room, keep your nurses back, maintain the horizontal position, and dare to wait. It is wonderful how long sometimes the insane will live without sleep, and still recover. Watch the strength; if that keeps up, and the tongue and mouth are not very dry, food is better than stimulants. You will not, however, send away all the brandy simply because the woman has recently been confined. Milk punch will sometimes give the sleep you are seeking. Convalescence will not necessarily follow sleep; sometimes there is a fair amount of sleep from the start, but the excitement goes on. In most of these cases I have considerable faith in bromide of potassium, in doses of twenty grains, three times daily. I often give it with compound tincture of cinchona where there is lack of strength.

After you get the sleep, remember that a little time is almost always needed before much or any improvement appears; but, no improvement showing after several days, you may then fairly feel that it is better to send to the hospital. Then state to the friends that the case is likely to be of some weeks' continuance, and when decided to remove, the sooner it is done the better. Your patient can probably travel with less risk than a week earlier, and you will have the consolation of having given the case a fair trial at home, and in many cases will, I think, have the satisfaction of seeing it recover there.

